# Circulating Cell-Free Tumor DNA in Advanced Pancreatic Adenocarcinoma Identifies Patients With Worse Overall Survival

**DOI:** 10.3389/fonc.2021.794009

**Published:** 2022-01-10

**Authors:** Gehan Botrus, Pedro Luiz Serrano Uson Junior, Puneet Raman, Adrienne E. Kaufman, Heidi Kosiorek, Jun Yin, Yu Fu, Umair Majeed, Mohamad Bassam Sonbol, Daniel H. Ahn, Isabela W. Chang, Leylah M. Drusbosky, Hiba Dada, Jason Starr, Mitesh Borad, Kabir Mody, Tanios S. Bekaii-Saab

**Affiliations:** ^1^ Division of Hematology and Oncology, Department of Medicine, Mayo Clinic, Scottsdale, AZ, United States; ^2^ Center for Personalized Medicine, Hospital Israelita Albert Einstein, Sao Paulo, Brazil; ^3^ Division of Oncology, Mayo Clinic, Jacksonville, FL, United States; ^4^ Guardant Health, Inc., Redwood City, CA, United States; ^5^ Center of individualized Medicine, Mayo Clinic, Rochester, MN, United States; ^6^ Mayo Clinic Cancer Center, Phoenix, AZ, United States

**Keywords:** circulating tumor DNA, ctDNA, KRAS, TP53, pancreatic cancer

## Abstract

**Background:**

Plasma-based circulating cell-free tumor DNA (ctDNA) genomic profiling by next-generation sequencing (NGS)is an emerging diagnostic tool for pancreatic cancer (PC). The impact of detected genomic alterations and variant allele fraction (VAF) in tumor response to systemic treatments and outcomes is under investigation.

**Methods:**

Patients with advanced PC who had ctDNA profiled at time of initial diagnosis were retrospectively evaluated. We considered the somatic alteration with the highest VAF as the dominant clone allele frequency (DCAF). ctDNA NGS results were related to clinical demographics, progression-free survival (PFS) and overall survival (OS).

**Results:**

A total of 104 patients were evaluated. Somatic alterations were detected in 84.6% of the patients. Patients with ≥ 2 detectable genomic alterations had worse median PFS (p < 0.001) and worse median OS (p = 0.001). *KRAS* was associated with disease progression to systemic treatments (80.4% vs 19.6%, p = 0.006), worse median PFS (p < 0.001) and worse median OS (p = 0.002). *TP53* was associated with worse median PFS (p = 0.02) and worse median OS (p = 0.001). The median DCAF was 0.45% (range 0-55%). DCAF >0.45% was associated with worse median PFS (p<0.0001) and median OS (p=0.0003). Patients that achieved clearance of *KRAS* had better PFS (p=0.047), while patients that achieved clearance of *TP53* had better PFS (p=0.0056) and OS (p=0.037).

**Conclusions:**

Initial detection of ctDNA in advanced PC can identify somatic alterations that may help predict clinical outcomes. The dynamics of ctDNA are prognostic of outcomes and should be evaluated in prospective studies.

## Introduction

Pancreatic ductal adenocarcinoma (PDAC) is the fourth leading cause of cancer-related deaths in the United States, with 60,430 estimated new cases and 48,220 expected related deaths in 2021 (1). In the world, it is expected 495,773 new cases would be diagnosed in 2020, ranked seventh as leading cancer-related deaths (2). Most patients are diagnosed with advanced stage disease and the 5-year overall survival probabilities remains poor even after several improvements to the treatment paradigm in recent years (1–4).

Interrogation of somatic and germline alterations by next-generation sequencing (NGS) in these tumors is proving to be important and impactful in the management of disease (5–7). Tissue NGS can delineate patients whose tumor has actionable biomarkers that could be treated with targeted agents, further improving outcomes (5). However, less than 10% of PDAC patients harbor an actionable somatic or germline biomarker, including microsatellite stability high (MSI-H), high tumor mutational burden (TMB), *BRCA1/2*, *BRAF* V600E, *KRAS* G12C, *HER2*, or activating fusions, generally observed in *KRAS* wild type tumors (8–11). Furthermore, obtaining tumor tissue for genomic analysis by NGS can be challenging considering the technical difficulties involved in the process of endoscopic ultrasound-guided tissue acquisition (12, 13). Furthermore, analysis of the biopsy samples can be complicated due to the presence of mixed desmoplastic stroma or insufficient tumor material, warranting repeated invasive procedures (13).

Liquid biopsies are non-invasive tests that can perform comprehensive genomic profiling from a blood sample. NGS of plasma-derived circulating cell-free tumor DNA (ctDNA) is being investigated as a potential tool for diagnosis and prognosis, and as alternative for tumor tissue in the identification of potential actionable biomarkers (14–18). ctDNA is shed from the tumor and metastatic lesions and exists in fragments in plasma, generated by lysis of tumor cells that have undergone apoptosis, necrosis cellular turnover (19, 20). Comparison of PDAC ctDNA and tissue NGS analysis showed high correlation and accuracy (21, 22). In localized PDAC, detection of *KRAS* and other mutations in ctDNA pre-operatively and post-operatively was related to worse recurrence-free survival and overall survival, with recurrence observed in all patients with detectable ctDNA post-surgery (23). In advanced PDAC, higher levels of ctDNA were associated with inferior overall survival, and several small retrospective cohorts suggest that mutations in *KRAS* detected by ctDNA are associated with worse specific disease outcomes (15, 24–27). The goal of this study was to evaluate ctDNA testing in PDAC patients at Mayo Clinic and to characterize the prognostic impact of mutated genes detected at diagnosis.

## Materials and Methods

### Patients

From December 2014 through October 2019, patients with PDAC underwent liquid biopsy testing using a clinically available assay (Guardant Health, Inc.). Two 10mL blood samples were obtained from patients cared for at Mayo Clinic in Florida and Arizona. In this study we evaluated 104 patients that had blood collected for ctDNA analysis at diagnosis of advanced disease. The data analysis from this patient cohort was reviewed and approved by the Mayo Clinic institutional review board.

### Comprehensive Genomic Testing in Plasma

All samples were shipped to Guardant Health, Inc., Redwood City, California as part of routine clinical care. After centrifugation of whole blood, 5 ng – 30 ng of cell -free DNA isolated from plasma was processed for digital NGS. The variant allele fraction (VAF) was calculated as the proportion of ctDNA harboring the variant in a background of wild type cell-free DNA (cfDNA). The assay demonstrated analytical sensitivity and specificity of 100% for single nucleotide variants >0.25% allele fraction (28, 29). Bioinformatics analysis of NGS data has been previously described. Most samples in this study were tested using a 73-gene panel.

Variant allele frequency (VAF) is a measurement of the percent of DNA fragments that harbor a somatic mutation (ctDNA) divided by the wild-type sequence derived from cell-free DNA (cfDNA). The dominant clone allele frequency (DCAF) is defined as the somatic alteration(s) detected in the sample with highest VAF, suggesting clonal alterations.

### Statistical Analysis

Continuous variables were compared between groups by Wilcoxon rank sum test, and Fisher Exact test was conducted for categorical data comparisons. The relationship between alteration types, such as mutation and amplification targetable status was evaluated using Spearman’s rank correlation. The analysis was conducted in 104 patients with baseline ctDNA results available prior to initiation of systemic therapy to determine the association of somatic alterations and disease stage (locally advanced or metastatic). Kaplan-Meier analysis was used to estimate progression-free survival (PFS) and overall survival (OS) based on stage or the absence/presence of somatic alterations. Patients without a progression/death event were considered censored at the date of last known follow-up in addition, Cox regression models were evaluated in a univariate and multivariable fashion. P values <0.05 were considered statistically significant. All computations were carried out in SAS version 9.3 and R version 3.6.2.

## Results

### Patient Demographics

A total of 104 patients were included in this study, 39 with stage III (locally advanced pancreatic ductal adenocarcinoma [LAPC]) and 65 with stage IV (metastatic disease[MPC]). Demographic data are summarized in **Table 1**. There was an equal number of male and female patients, of which 66 patients underwent chemotherapy with gemcitabine and paclitaxel (64%) and 29 patients with FOLFIRINOX (28%). The location of the pancreatic mass differed significantly (p=0.02) between groups, as 61.5% of patients with LAPC had tumors located in the pancreatic head. Among patients with MPC, 33.8% had tumors located in the pancreatic head, 29.2% in the body, and 27.7% in the tail. Additional differences in genetic alterations between LAPC and MPC are presented in supplement (**Supplementary Tables 1** **–** **3** and **Supplementary Figure 1**).

**Table 1:** Demographic table.

	Locally advanced (N = 39)	Metastatic (N = 65)	Total (N = 104)	p value
**Age**				
Median	71.0 (43-87)	70.0(50-91)	70.0 (43-91)	
**Sex**				
Female	20 (51.3%)	32 (49.2%)	52 (50.0%)	
Male	19 (48.7%)	33 (50.8%)	52 (50.0%)	
**Chemotherapy**				
FOLFIRINOX	16 (41.0%)	13 (20.0%)	29 (27.9%)	
Gem+Abraxane	22 (56.4%)	44 (67.7%)	66 (63.5%)	
No chemotherapy	1 (2.6%)	7 (10.8%)	8 (7.7%)	
pembrolizumab	0 (0.0%)	1 (1.5%)	1 (1.0%)	
**Overall Response rate (CR+PR+SD)**				**<0.001^2^**
No. missing	4	10	14	
No	12 (34.3%)	42 (76.4%)	54 (60.0%)	
Yes	23 (65.7%)	13 (23.6%)	36 (40.0%)	
**Vital status**				**0.006^2^**
Alive	22 (56.4%)	19 (29.2%)	41 (39.4%)	
Dead	17 (43.6%)	46 (70.8%)	63 (60.6%)	
**CA19-9 (U/mL)**				**<0.001^1^**
Count	35	52	87	
Median	251.0 (0.0-3564)	1267.0 (1.0-1800000)	774.0 (0.0-1800000)	
**Follow up time (months)**				**0.075^1^**
Count	22	19	41	
Median	17.7 (1.2-40.1)	10.1 (2.0-33.7)	12.9 (1.2-40.1)	

CR, complete response; PR, partial response; SD, stable disease.

^1^ANOVA F-test p-value.

^2^Chi-Square p-value.

A total of 23 patients had blood collected for NGS at diagnosis of advanced disease and upon disease progression to first line SOC therapy.

Patients with LAPC had higher overall response rate to standard of care compared to MPC (65.7% vs 23.6%, respectively; p<0.001). Interestingly, 83.1% of patients with somatic alterations detected *via* liquid biopsy had initial liver metastasis compared to 40% of patients without any detected alterations (p <0.001) (**Supplementary Figure 2**).

### Genomic Landscape of ctDNA in PDAC

Ninety-one percent of MPC had at least one genetic alteration (n=59) compared to 74% of patients with LAPC (n=29; p=0.03). Seventy five percent of MPC patients had at least 2 alterations (n=49) compared to 36% with LAPC (n=14; p<0.001). The median number of detectable somatic alterations was 3 in MPC compared to 1 in LAPC.

*KRAS* mutations were detected in 73.8% of MPC compared to 43.6% of LAPC (p=0.002). Approximately 66% of patients with MPC harbored 1 *KRAS* mutation and 7.7% harbored 2 *KRAS* mutations, compared to 43.6% and 0% for those with LAPC (p=0.004). Additionally, 85% of patients with MPC who harbored a *KRAS* mutation experienced the liver as the primary site of metastasis compared to 62% of patients without *KRAS* mutations (p=0.03). *TP53* mutations were also detected more frequently in patients with metastases, detected in 69% of patients with MPC compared to 43.6% with LAPC (p=0.01) (**Supplementary figure 2**, **3**). Similarly, to *KRAS*, 21.5% of patients with MPC had at least 2 mutations in *TP53* compared to 5% of LAPC (p=0.01). Other gene alterations with significant differences include *SMAD*, which was present in 13.8% of MPC compared to 0% of LAPC (p=0.02) (**Supplementary Figure 1**). Overall, *KRAS* and *TP53* were the two most frequent alterations followed by *CCND2*, *BRCA1/2* or *ATM*, and *SMAD* (**Supplementary Table 1**). On multivariate analysis detection of KRAS and metastatic disease were statistically associated with worse PFS (**Supplementary Table 2**). On multivariate analysis for OS, metastatic disease was statistically associated with worse overall survival (**Supplementary Table 3**). DCAF >0.45% remained statistically associated with inferior PFS and OS in a regression analysis with disease status (**Supplementary Table 4**).

### Somatic Alterations as a Prognostic Tool

Of the 63 patients who did not respond to treatment, 71.4% harbored a *KRAS* mutation compared to 40.7% of patients who did achieve a favorable response (p=0.006). Of the non-responders, 66.7% had one *KRAS* mutation while 5% had at least 2 mutations in *KRAS*. In patients who responded to therapy, those numbers decreased to 37% and 3.7% respectively (p=0.22).

**Figure 1:**
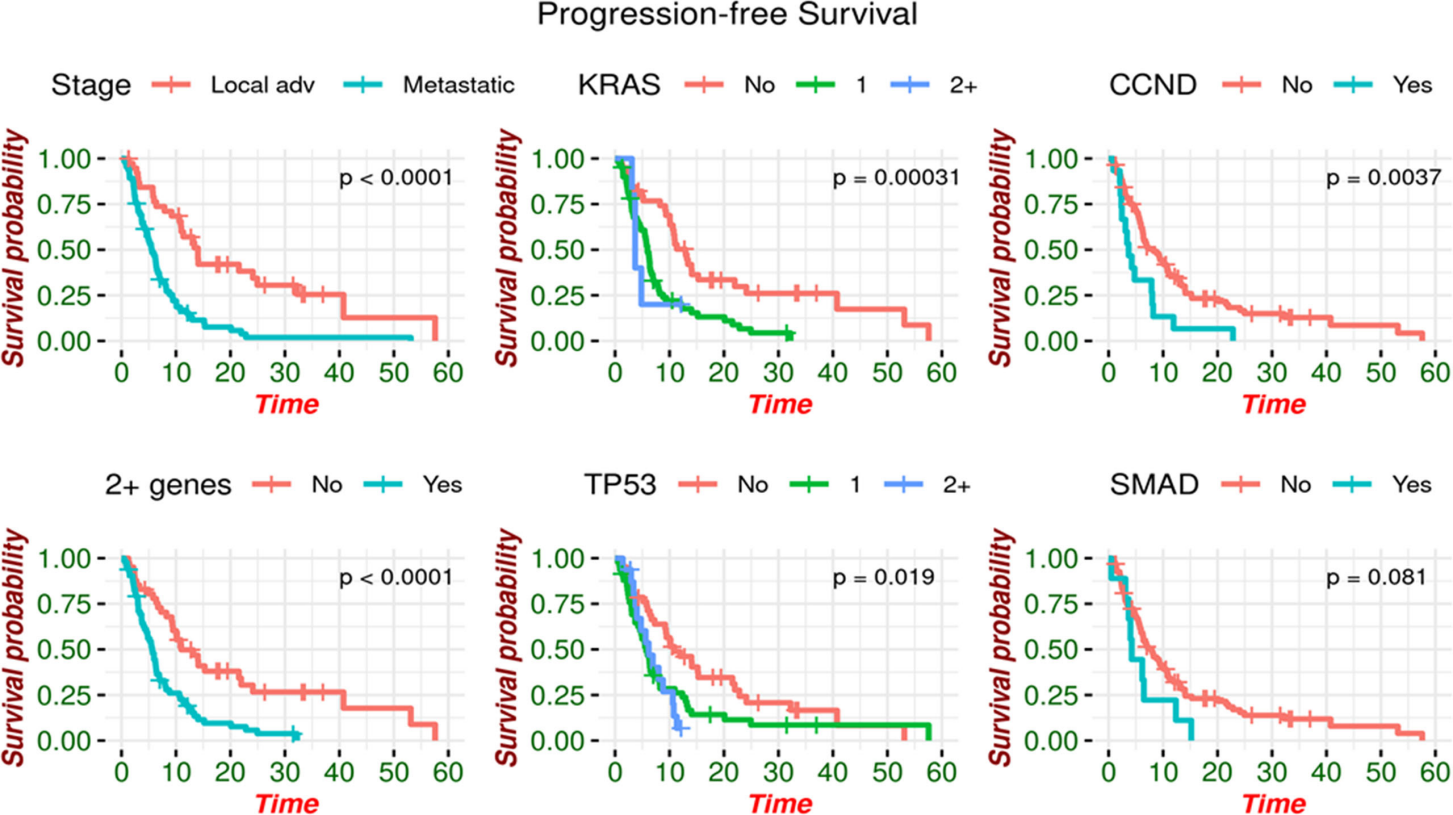
Progression-free survival by genomic alteration. PFS is increased in patients with no somatic alterations detected.

Additional metrics of prognostication has demonstrated an association between the presence of these gene mutations and poor outcomes. Patients with LAPC had a median PFS of 14.0 months (95% CI: 10.9 - 32.2) compared to 5.5 months (95% CI: 4.6 - 6.9) in those with metastatic disease (p<0.0001). PFS significantly increased with a median of 15.3 months (95% CI: 10.1 – Not estimated) in patients with no somatic alterations detected *via* liquid biopsy compared to 6.2 months (95% CI: 5.4 - 8.0) in those with somatic alterations detected (p=0.005, **Figure 1**). Specifically, those with <2 somatic alterations had a significantly increased median PFS of 11.0 months (95% CI: 9.3 - 24.1) compared to 5.6 months (95% CI: 4.2 - 6.9) in those with ≥ 2 alterations detected. Amongst patients with MPC, those with ≥ 2 alterations had a significantly decreased median PFS of 5.2 months (95% CI: 3.7 - 6.3) compared to 8.2 months (95% CI: 5.0 - 15.3) in those with <2 or no alterations.

Patients with *CCND2* mutations had a significantly reduced median PFS of 3.7 months (95% CI: 2.4 - 8.2) compared to 8.2 months in those without a mutation in *CCND2* (95% CI: 6.3 – 11.0). Those with *KRAS* mutations also experienced a statistically significant reduction, with median PFS of 5.8 (95% CI: 4.6 - 6.7) for *KRAS* mutant as compared to 12.9 months (95% CI: 10.1 – 22.0) for *KRAS* not being detected. The reduction in median PFS was more pronounced when analyzing the number of mutations, as those with two or more *KRAS* mutations had a median PFS of 3.7 months (95% CI: 3.68 – Not estimated) compared to 5.9 months with 1 alteration (95% CI: 4.8 - 6.9) and 12.9 months with no *KRAS* alteration detected (95% CI: 10.1 – 22.0). In patients with *TP53* mutations, median PFS was also significantly reduced to 5.9 months (95% CI: 4.8 - 7.9) compared to 10.9 months for patients without *TP53* mutations (95% CI: 9.2 – 22.0) (**Figure 1**).

**Figure 2:**
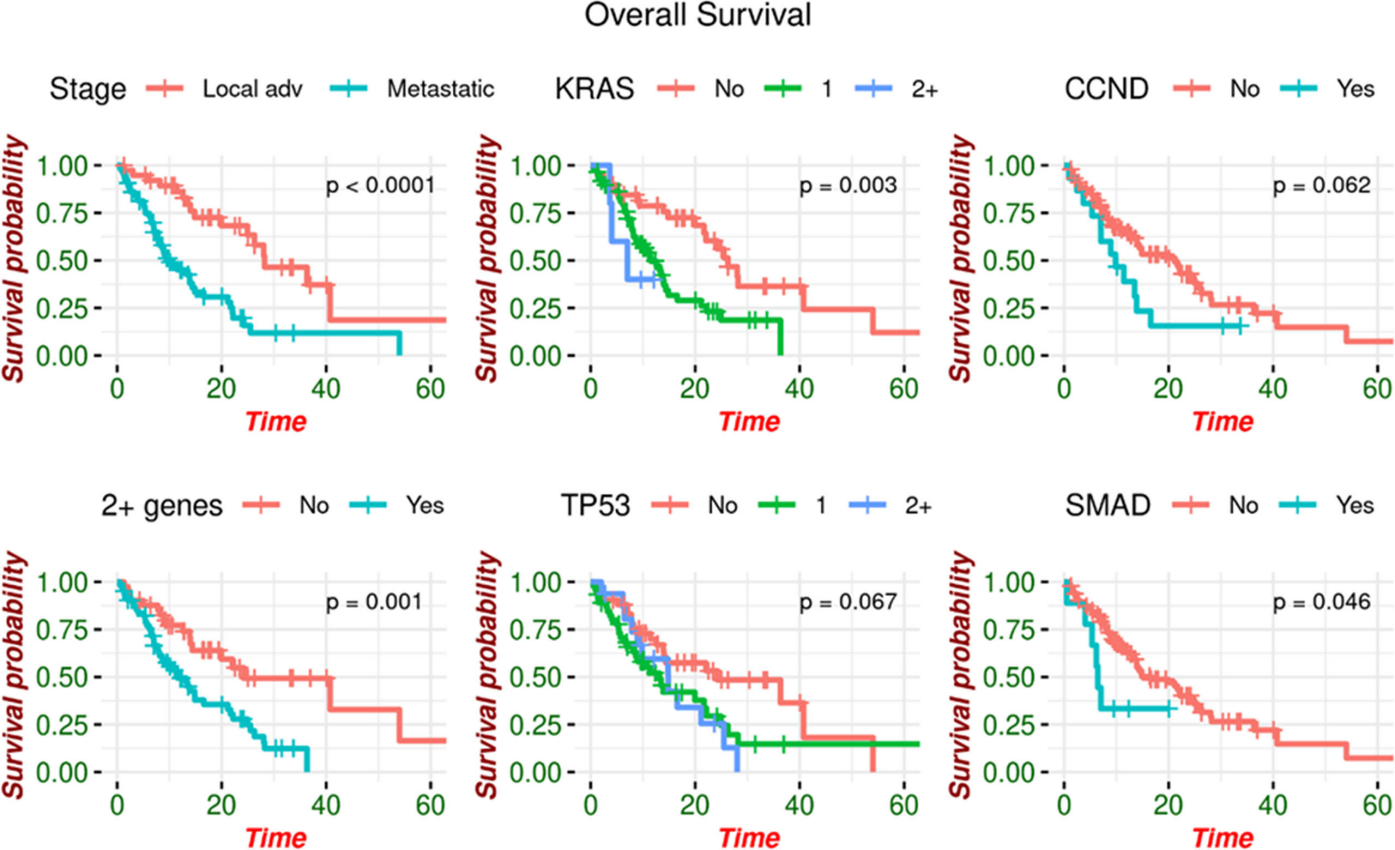
Overall survival by genomic alteration. OS is increased in patients with no somatic alterations detected.

Patients with at least two somatic alterations had a lower median overall survival (OS) of 11.5 months (range 8.11 - 21.1 months) compared to 24.2 months (95% CI: 14.38 – Not estimated) in patients with ≤ 1 alteration. This association was preserved when separately analyzing patients with MPC, as those with ≥2 alterations had a median OS of 9.82 months (95% CI: 7.03 - 16.6) compared to 13.89 months (95% CI: 7.78 – Not estimated) in patients with ≤1 alteration. This was also noted in LAPC, with median OS of 24.9 months (95% CI: 40.8 – Not estimated) for patients with ≥2 alterations and 40.8 months (95% CI: 13.5 – Not estimated) for patients with ≤1 alteration. Patients with and without a *KRAS* mutation had median OS of 11.5 months (95% CI: 8.21 - 14.8) and 26.3 months (95% CI: 21.67 – Not estimated) respectively. Similarly, those with and without *TP53* mutations had median OS of 13.5 months (95% CI: 9.06 - 21.7) and 24.2 months (95% CI: 14.02 – Not estimated) (**Figure 2**).

**Figure 3:**
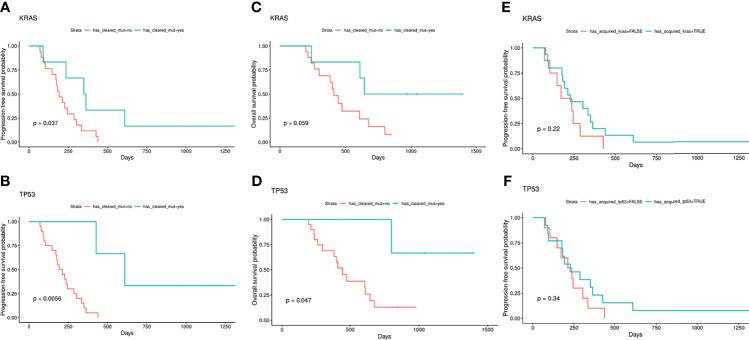
TP53 and KRAS with serial testing. Patients with clearance at any timepoint of KRAS (6/18) 33% and/or *TP53* (3/18)17% achieved improved PFS [p=0.037; p=0.0056, respectively **(A, B)**]. Clearance of *KRAS* in ctDNA after first line SOC trended towards improved OS (p=0.059) **(C)**, while clearance of *TP53* significantly improves OS (p=0.047) **(D)**, though in a small sample size. If a patient is found to have a mutation in *TP53* or *KRAS* upon disease progression, (12/23) 50% acquired TP53 or KRAS mutations on progression; (10/23) 43.5% patients acquired TP53, (8/23) 35% patients acquired KRAS mutations (some patients acquired both mutations), PFS is not significantly impacted **(E, F)**. **(A)** KRAS clearance and progression-free survival; **(B)** TP53 clearance and progression-free survival; **(C)** KRAS clearance and overall survival; **(D)** TP53 clearance and overall survival; **(E)** KRAS acquisition and progression-free survival; **(F)** TP53 acquisition and progression-free survival.

Changes in molecular profiles from baseline to progression were analyzed for overall survival and progression free survival in 23 patients. Eighteen (78%) samples harbored *TP53* and/or *KRAS* alterations at baseline. Patients with clearance at any timepoint of *TP53* (3/18)17% and/or KRAS (6/18) 33% achieved improved PFS (p=0.0056; p=0.037, respectively). Clearance of *KRAS* in ctDNA after first line SOC trended towards improved OS (p=0.059), while clearance of *TP53* significantly improves OS (p=0.047), though in a small sample size. Interestingly, if a patient is found to have a mutation in *TP53* or *KRAS* upon disease progression, (12/23) 50% acquired TP53 or KRAS mutations on progression; (10/23) 43.5% patients acquired TP53, (8/23) 35% patients acquired KRAS mutations (some patients acquired both mutations), PFS is not significantly impacted (**Figure 3**).

### Variant Allele Fraction (VAF) as a Prognostic Tool

**Figure 4:**
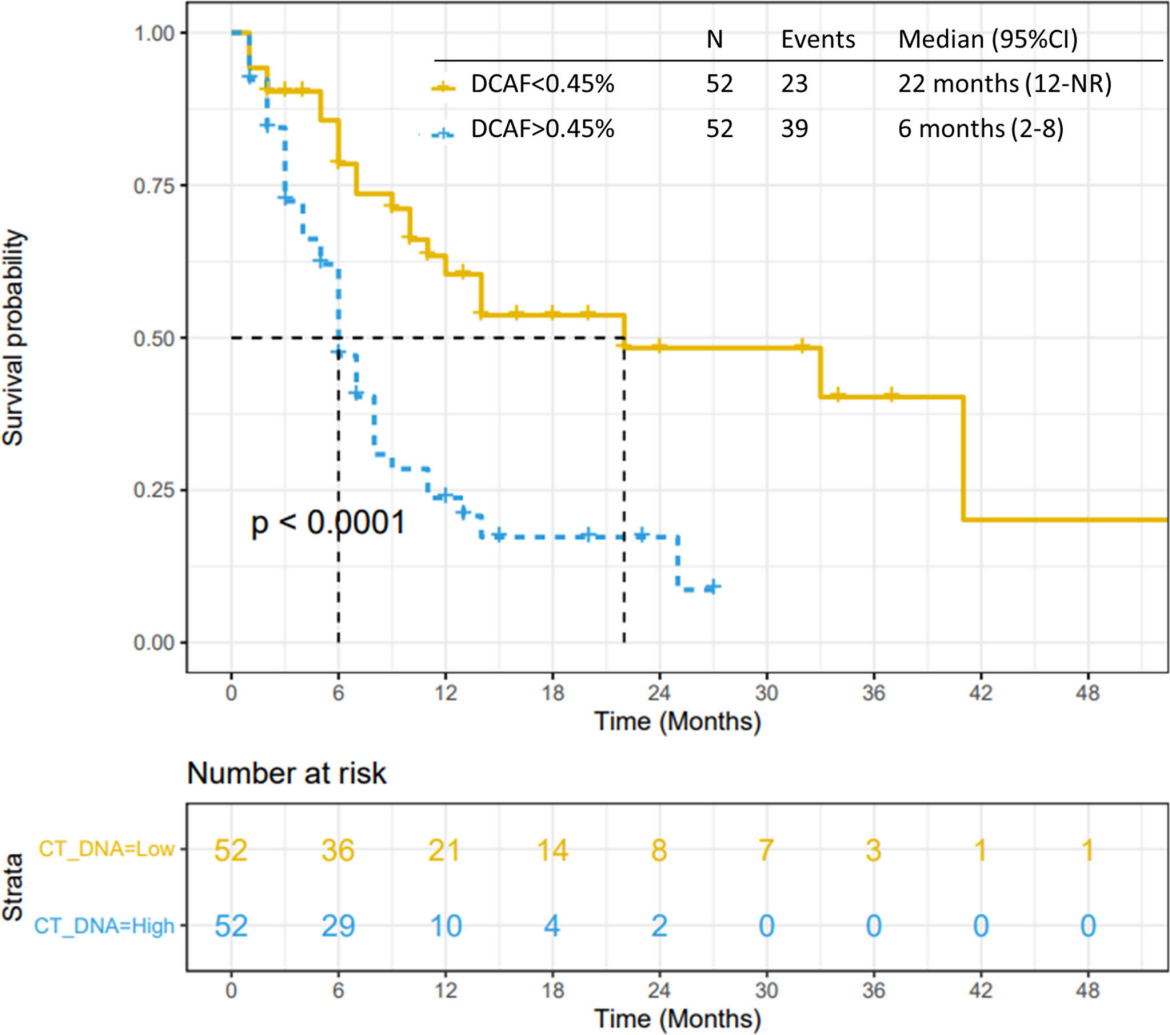
Progression-free survival by DCAF. Patients with dominant clone allele frequency > 0.45% have worse PFS.

**Figure 5:**
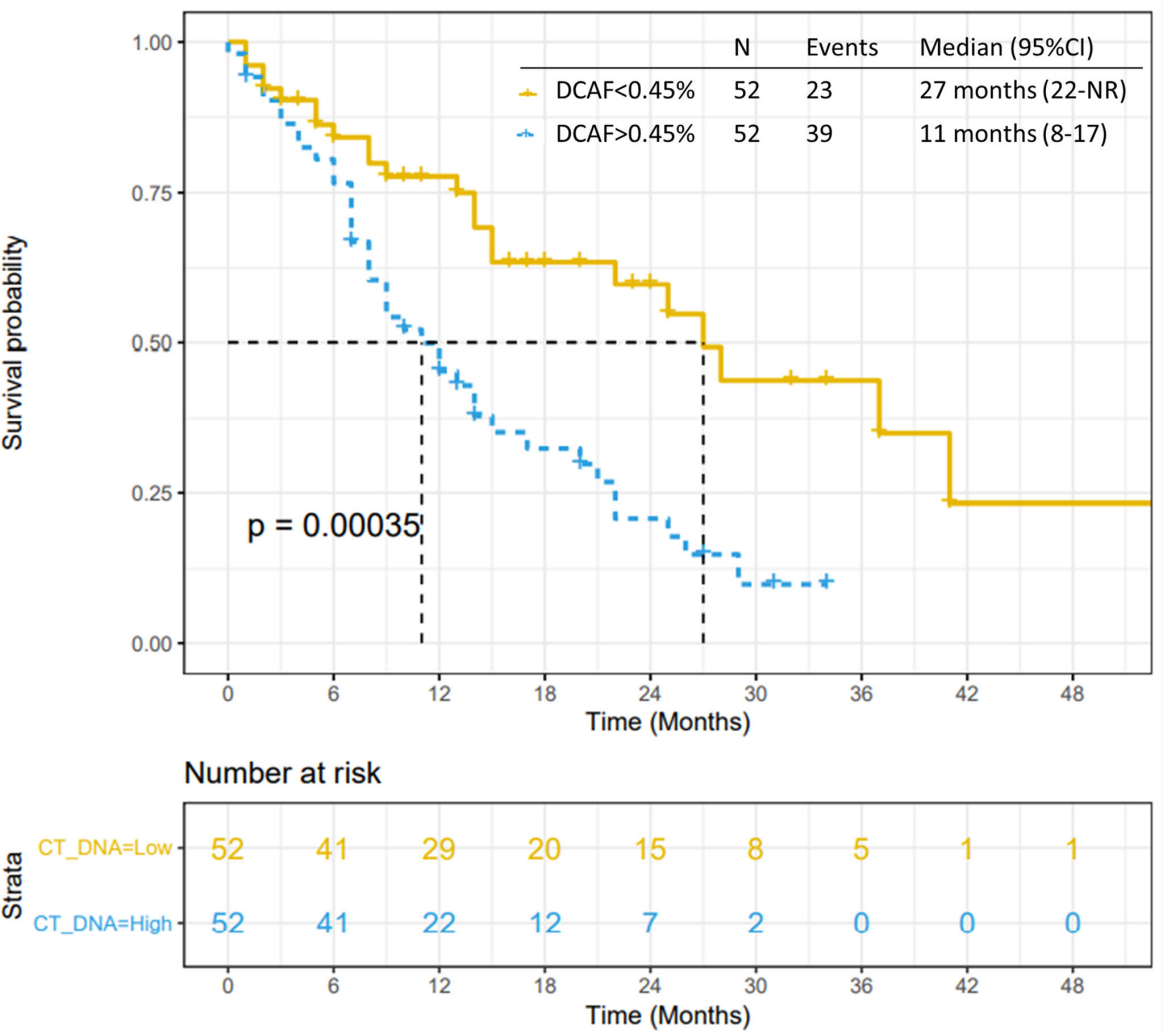
Overall survival by DCAF. Patients with dominant clone allele frequency > 0.45% have worse OS.

All 104 patients were included in the VAF analysis. The median dominant clone allele frequency (DCAF) was 0.45% (range 0-55%). The presence of DCAF >0.45% was associated with worse median PFS (p<0.0001; **Figure 4**) and median OS (p=0.0003; **Figure 5**). However, DCAF was not associated with co-occurring *KRAS* mutations (p=0.52).

VAF was not statistically associated with tumor response to systemic treatments (**Supplementary Figures 4**, **5**). However, DCAF >0.45% in 66 patients treated with gemcitabine and nab-paclitaxel was statistically associated with worse PFS (p<0.0001) and OS (p=0.0007). DCAF >0.45% was not statistically associated with inferior outcomes in patients treated with FOLFIRINOX, however caution should be made in this sub-analysis considering small sample size (29 patients).

## Discussion

This study shows that mutations in *KRAS*, *TP53* and *CCND2*, along with the VAF detected by liquid biopsy testing at diagnosis could be recognized as prognostic biomarkers in patients with advanced pancreatic cancer. Furthermore, this study demonstrates that patients who harbor multiple somatic alterations in ctDNA have a worse median overall survival than those who have one or no somatic alterations.

*KRAS* mutations are considered one of the initiating genomic processes in the development of pancreatic cancer, causing permanent activation of RAS pathway leading to carcinogenesis and resistance to systemic treatments (30–32). Studies previously showed that *KRAS* wild-type PDAC exhibits distinct features, including improved disease specific outcomes such as overall survival and more favorable response to systemic treatments (33, 34). In a total of 104 patients with advanced

PDAC, we detected a significantly higher number of genetic alterations in patient with MPC as compared LAPC. *KRAS* mutations were detected in 73.8% of MPC compared to 43.6% of LAPC.

*KRAS* mutations detected in ctDNA were more frequently identified in patients that did not respond to chemotherapy, and those with KRAS alterations demonstrated inferior median progression-free survival (5.78 vs 12.94 months) and inferior median overall survival (11.5 vs 26.3 months) when compared to *KRAS* non-detected patients. Our observation, combined by findings from other groups, corroborates the hypothesis that detection of ctDNA mutated *KRAS* at diagnosis of advanced pancreatic cancer is correlated with reduced time-to-progression and overall survival (35–37). Patients with a *KRAS* mutation more frequently presented the liver as the primary site of metastasis compared to those without *KRAS* mutations, several studies indicate that liver metastasis confers worse overall survival probabilities in MPC when compared to other metastatic sites such as the lung or bones (38, 39), and further prospective analyses in larger cohorts would be necessary to address those associations. Interestingly, in a subgroup of patients, clearance of *TP53* 17% (3/18)or *KRAS* 33% (6/18) mutations after chemotherapy treatment was associated with improved PFS (p=0.0056 and p=0.037, HR of 0.087 and 0.32, respectively), and this observation is corroborated by other groups and highlights additional clinical utility of ctDNA in PDAC (27, 35). In the future, the detection of *KRAS* in ctDNA could be used as monitoring strategy during systemic treatment of advanced disease, were the dynamics of ctDNA measured during chemotherapy cycles with FOLFIRINOX or gemcitabine-based regimens could be a predictor of disease progression or an early indicator of response, and as a surrogate of metastatic burden, to provide additional prognostication at the time of, or even before, computerized tomography scans or CA 19.9 (36, 40, 41).

In PDAC, alterations in *TP53* are one of the most common mutations, with 50-70% of PDAC samples harboring somatic *TP53* mutations (6, 30, 42). Deep whole-exome sequencing revealed that *TP53* mutations were also correlated to a basal-like subgroup, a subtype of PDAC correlated with worse overall survival and poor response to chemotherapy (30, 43, 44). In this cohort, *TP53* mutations were also predominantly detected in MPC (69.2%) compared to LAPC (43.6%). Furthermore, median progression-free survival (5.94 vs 10.9 months, respectively) and median overall survival (13.5 to 24.2 months, respectively) was also significantly reduced in patients with *TP53* mutations in ctDNA. Considering that the most frequently mutated genes were *KRAS* and *TP53*, as expected, a higher median PFS was observed in those patients who had no somatic alterations detected in ctDNA compared to those with alterations (15.27 versus 6.24 months), and this remained consistent for patients with zero or no genetic alterations detected in ctDNA compared to those with at least 2 alterations (10.97 versus 5.62 months, respectively). This result highlights that ctDNA and detection of *KRAS* and *TP53* could be used as a stratification tool to guide prospective studies in advanced PDAC.

In tissue samples, somatic alterations in *SMAD4* are detected in about 20-30% of patients with PDAC (45). Mutations in *SMAD4* are related to advanced disease, poor overall survival, and recurrence after localize treatment in resectable pancreatic cancer (46). Although no association with progression-free survival or overall survival were detected in our cohort, a higher rate of *SMAD4* mutations were detected in MPC (13.8%) compared to LAPC (0%). Larger cohorts evaluating SMAD4 detection by ctDNA at diagnosis and between treatments would be necessary to address the real impact of this biomarker as monitoring strategy or prognostic factor. CCND2 (cyclin D2) regulates CDK kinases, forms a complex with CDK4 and CDK6, and possesses multiple functions necessary for cell cycle G1/S transition (47). Genomic alterations in *CCND2* are reported in multiple malignances including renal cell carcinoma (48) and colon cancer (49) and *CCND2* overexpression is related to poor overall survival in patients with gastric cancer (50). Although only 14% of the patients included in this study had *CCND2* mutations detected in ctDNA, the presence of a *CCND2* mutation was statistically associated with poor median progression-free survival when compared to patients with no detectable mutations [3.6 versus 8.2 months, respectively (p=0.0037)].

Lastly, in this study we evaluated variant allelic frequencies. Variant allele frequency changes between treatments and is related to outcomes in pancreatic cancer (24, 25). In an analysis of 94 patients with advanced PC utilizing ctDNA testing with the same platform of our study, total %ctDNA ≥ 0.6% was associated with worse median overall survival (6.3 months versus 11.7 months, p=0.001). However, maximum ctDNA of 0.4% was not associated with worse outcomes (24). In our analysis, median of the highest VAF was 0.45% and is significantly associated with worse PFS and OS. These results reinforce that VAF and DCAF can be used as a stratification tool, however the cutoff value to be used should be carefully evaluated in larger cohorts or prospective trials.

There are several limitations in this study. The timing of ctDNA analysis was not consistent across all patients and it would be necessary to evaluate ctDNA prospectively at diagnosis and between chemotherapy treatments in larger cohorts to fully understand the prognostic and predictive utility of the platform. Also, in patients where *KRAS* or *TP53* were not detected, there is a chance that a mutation was present but below the limit of detection for the ctDNA assay. For patients who had no alterations detected by liquid biopsy, tumor shed may have been suppressed by therapy, the patient may have indolent of slow-growing disease or low disease burden, or the tumor is shedding very low amounts of ctDNA below the level of detection of the assay. Patients who had higher numbers of genomic alterations detected were related to worse PFS and OS. It would be necessary to address *KRAS* and *TP53* to fully understand the impact of specific mutations identified and whether they contributed a distinct impact on clinical outcomes. In multivariate analysis detection of specific genetic mutations did not translate in worse overall survival, metastatic disease and DCAF>0.45% remained associated with inferior PFS and OS. It has been already shown and discussed that KRAS, TP53, and SMAD4 are the main genetic findings in pancreatic cancer and considering that most patients will have these genomic alterations it would be necessary bigger samples of patients to identify impact of the absence of these mutations in outcomes. Considering the findings, VAF could serve as a stratification factor for advanced pancreatic cancer with detectable somatic mutations in ctDNA.

## Conclusion

This study suggests that evaluation of ctDNA at diagnosis of advanced PDAC is a prognostic tool that may be applicable to clinical practice. This evaluation may be incorporated for deciding therapeutic strategies, designing, and enrolling patients onto clinical trials, and as an alternative genotyping assay in cases where tumor tissue samples are scarce or hardly obtainable. Perhaps a more important and impactful application would be utilizing the changes in ctDNA to guide early switch in systemic therapy, both in the neoadjuvant setting and MPC.

## Data Availability Statement

The original contributions presented in the study are included in the article/**Supplementary Materials**. Further inquiries can be directed to the corresponding author.

## Ethics Statement

The studies involving human participants were reviewed and approved by IRB Mayo Clinic Arizona. Written informed consent for participation was not required for this study in accordance with the national legislation and the institutional requirements.

## Author Contributions

GB and PU has written the manuscript and created the tables, review, assisted in the design, and final approval of the manuscript. PR, AK, HK, JY, YF, UM, MS, DA, IC, LD, HD, JS, MB, KM, and TB-S assisted in the design, review, and final approval of the manuscript. TB-S assisted in project administration and conceptualization. All authors contributed to the article and approved the submitted version.

## Funding

The Mayo Clinic Hepatobiliary SPORE (P50CA 210964) funded the statistical analysis for this project.

## Conflict of Interest

Authors YF, LD and HD were employed by company Guardant Health, Inc.

The remaining authors declare that the research was conducted in the absence of any commercial or financial relationships that could be construed as a potential conflict of interest.

## Publisher’s Note

All claims expressed in this article are solely those of the authors and do not necessarily represent those of their affiliated organizations, or those of the publisher, the editors and the reviewers. Any product that may be evaluated in this article, or claim that may be made by its manufacturer, is not guaranteed or endorsed by the publisher.
