# Supplementary material for: Circulating Cell-Free Tumor DNA in Advanced Pancreatic Adenocarcinoma Identifies Patients With Worse Overall Survival
Source: Front Oncol. 2022 Jan 10;11:794009. doi: 10.3389/fonc.2021.794009 (PMC8784799; doi:10.3389/fonc.2021.794009)
Supplement: Supplementary file 1 [file DataSheet_1.docx]

**Supplementary files:**

**Supplementary Table 1: Association of genetic alterations to disease stage**

**Supplementary Table 2: Progression free survival univariate and multivariable analysis**

**Supplementary Table 3: Overall survival univariate and multivariable analysis**

**Supplementary Table 4: PFS and OS regression analysis of DCAF>0.45% and disease status**

**Supplementary Figure 1: Distribution of Biomarkers by Disease Stage – Locally Advanced vs Metastatic**

**Supplementary Figure 2: KRAS and TP53 variants in metastatic sites**

**Supplementary Figure 3: Disease Stage Association with Somatic Alterations**

**Supplementary Figure 4: ctDNA and response rate**

**Supplementary Figure 5: ctDNA and disease control rate**

**Supplementary Table 1: Association of genetic alterations to disease stage**

|  | **Locally advanced**  **(n=39)** | **Metastatic**  **(n=65)** | **Total**  **(n=104)** | **p-value** |
| --- | --- | --- | --- | --- |
| BRCA1/2 or ATM |  |  |  | 0.459 |
| Absent | 36 (92.3%) | 57 (87.7%) | 93 (89.4%) |  |
| Present | 3 (7.7%) | 8 (12.3%) | 11 (10.6%) |  |
| CCND2 |  |  |  | 0.037 |
| Absent | 37 (94.9%) | 52 (80.0%) | 89 (85.6%) |  |
| Present | 2 (5.1%) | 13 (20.0%) | 15 (14.4%) |  |
| SMAD |  |  |  | 0.015 |
| Absent | 39 (100.0%) | 56 (86.2%) | 95 (91.3%) |  |
| Present | 0 (0.0%) | 9 (13.8%) | 9 (8.7%) |  |
| KRAS |  |  |  | 0.002 |
| Absent | 22 (56.4%) | 17 (26.2%) | 39 (37.5%) |  |
| Present | 17 (43.6%) | 48 (73.8%) | 65 (62.5%) |  |
| KRAS category |  |  |  | 0.004 |
| Absent | 22 (56.4%) | 17 (26.2%) | 39 (37.5%) |  |
| Present - 1 | 17 (43.6%) | 43 (66.2%) | 60 (57.7%) |  |
| Present - 2 | 0 (0.0%) | 5 (7.7%) | 5 (4.8%) |  |
| TP53 |  |  |  | 0.010 |
| Absent | 22 (56.4%) | 20 (30.8%) | 42 (40.4%) |  |
| Present | 17 (43.6%) | 45 (69.2%) | 62 (59.6%) |  |
| TP53 category |  |  |  | 0.013 |
| Absent | 22 (56.4%) | 20 (30.8%) | 42 (40.4%) |  |
| Present - 1 | 15 (38.5%) | 31 (47.7%) | 46 (44.2%) |  |
| Present - 2 | 2 (5.1%) | 14 (21.5%) | 16 (15.4%) |  |
| PIK3CA |  |  |  | 0.447 |
| Absent | 35 (89.7%) | 61 (93.8%) | 96 (92.3%) |  |
| Present | 4 (10.3%) | 4 (6.2%) | 8 (7.7%) |  |
| ATM |  |  |  | 0.613 |
| Absent | 37 (94.9%) | 60 (92.3%) | 97 (93.3%) |  |
| Present | 2 (5.1%) | 5 (7.7%) | 7 (6.7%) |  |
| Presence of genes alterations |  |  |  | < 0.001^2^ |
| No alterations | 16 (41.0%) | 8 (12.3%) | 24 (23.1%) |  |
| Yes alterations | 23 (59.0%) | 57 (87.7%) | 80 (76.9%) |  |
| Number of genetic alterations |  |  |  | < 0.001^1^ |
| Count | 39 | 65 | 104 |  |
| Median | 1.0 (0.0-6.0) | 3.0 (0.0-12.0) | 2.0 (0.0-12.0) |  |
| **2+ genetic alterations** |  |  |  | < 0.001^2^ |
| No | 25 (64.1%) | 16 (24.6%) | 41 (39.4%) |  |
| Yes | 14 (35.9%) | 49 (75.4%) | 63 (60.6%) |  |

**Supplementary Table 2: Progression free survival univariate and multivariable analysis**

| Variables | Group | Univariate HR (95%CI) | P Value | Multivariable HR *(95% CI) | P value |
| --- | --- | --- | --- | --- | --- |
| Genetic Alterations | Yes vs No | 2.45 (1.29, 4.65) | 0.006 |  |  |
|  | <2 or ≥2 | 2.63 (1.65, 4.20) | < 0.001 | 1.04 (0.47, 2.29) | 0.90 |
|  |  |  |  |  |  |
| CCND | Yes vs No | 2.26 (1.28, 3.99) | 0.005 | 1.35 (0.71, 2.54) | 0.40 |
| SMAD | Yes vs No | 1.84 (0.92, 3.71) | 0.086 | 0.95 (0.45, 1.98) | 0.90 |
| KRAS | 1 vs No | 2.51 (1.56, 4.04) | < 0.001 | 2.00 (1.03, 3.87 | 0.039 |
|  | ≥2 vs No | 2.99 (1.03, 8.72) | 0.045 | 5.04 (1.34, 19.0) | 0.017 |
| TP53 | 1 vs No | 1.75 (1.10, 2.79) | 0.018 | 1.33 (0.72, 2.45) | 0.40 |
|  | ≥2 vs No | 2.14 (1.12, 4.10) | 0.021 | 2.04 (0.91, 4.56) | 0.083 |
| PIK3CA | Yes vs No | 1.19 (0.57, 2.48) | 0.637 | 1.71 (0.65-4.48) | 0.3 |
| ATM | Yes vs No | 0.56 (0.20, 1.53) | 0.255 | 0.84 (0.17-4.17) | 0.8 |
| Disease | MPC vs LAPC | 3.16 (1.95, 5.11) | <0.001 | 2.65 (1.54, 4.56) | <0.001 |

**Supplementary Table 3: Overall survival univariate and multivariable analysis**

| Variables | Group | Univariate HR (95%CI) | P Value | Multivariable HR (95% CI) | P value |
| --- | --- | --- | --- | --- | --- |
| Genetic Alterations | Yes vs No | 2.63 (1.13, 6.13) | 0.026 |  |  |
|  | <2 or ≥2 | 2.55 (1.43, 4.54) | 0.002 | 0.87 (0.33, 2.31) | 0.80 |
|  |  |  |  |  |  |
| CCND | Yes vs No | 1.81 (0.96, 3.43) | 0.067 | 1.14 (0.55, 2.37) | 0.70 |
| SMAD | Yes vs No | 2.35 (0.99, 5.57) | 0.053 | 1.42 (0.57, 3.50) | 0.50 |
| KRAS | 1 vs No | 2.49 (1.40, 4.43) | 0.002 | 1.75 (0.81, 3.82) | 0.20 |
|  | ≥2 vs No | 3.92 (1.11, 13.85) | 0.034 | 2.03 (0.50, 8.26) | 0.30 |
| TP53 | 1 vs No | 1.82 (1.04, 3.20) | 0.038 | 1.87 (0.91, 3.85) | 0.087 |
|  | ≥2 vs No | 1.94 (0.95, 3.98) | 0.071 | 1.52 (0.62, 3.73) | 0.40 |
| PIK3CA | Yes vs No | 1.10 (0.33, 2.59) | 0.495 | 2.00 (0.59-6.76) | 0.3 |
| ATM | Yes vs No | 0.92 (0.33, 2.59) | 0.502 | 0.78 (0.14-4.28) | 0.8 |
| Disease | MPC vs LAPC | 3.35 (1.86, 6.02) | <0.001 | 2.82 (1.45, 5.51) | 0.002 |

**Supplementary Table 4: PFS and OS regression analysis of DCAF>0.45% and disease status**

| PFS | Hazard Ratio | 95%CI | P value |
| --- | --- | --- | --- |
| MPC vs LAPC | 2.37 | [ 1.26 , 4.44 ] | 0.007 |
| DCAF>0.45% | 2.15 | [ 1.19 , 3.87 ] | 0.01 |

| OS | Hazard Ratio | 95%CI | P value |
| --- | --- | --- | --- |
| MPC vs LAPC | 2.3 | [ 1.23 , 4.29 ] | 0.009 |
| DCAF>0.45% | 1.88 | [ 1.04 , 3.38 ] | 0.03 |

**Supplementary Figure 1: Distribution of Biomarkers by Disease Stage – Locally Advanced vs Metastatic**

**
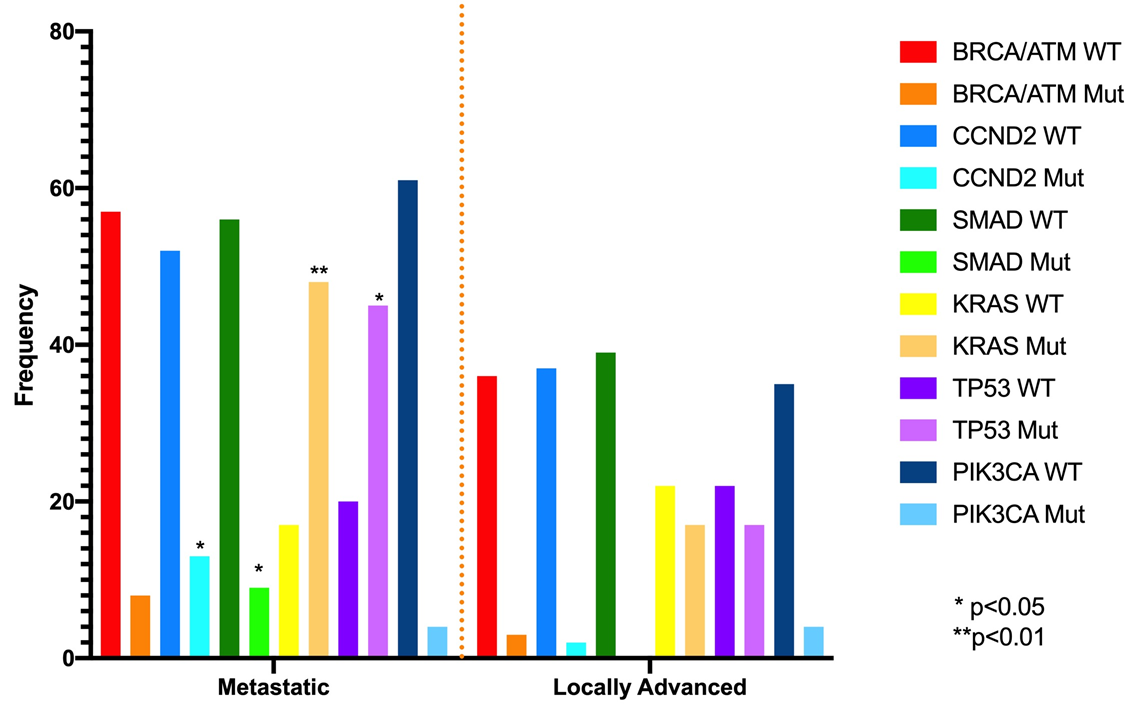
**

**Supplementary figure 2: KRAS and TP53 variants in metastatic sites**


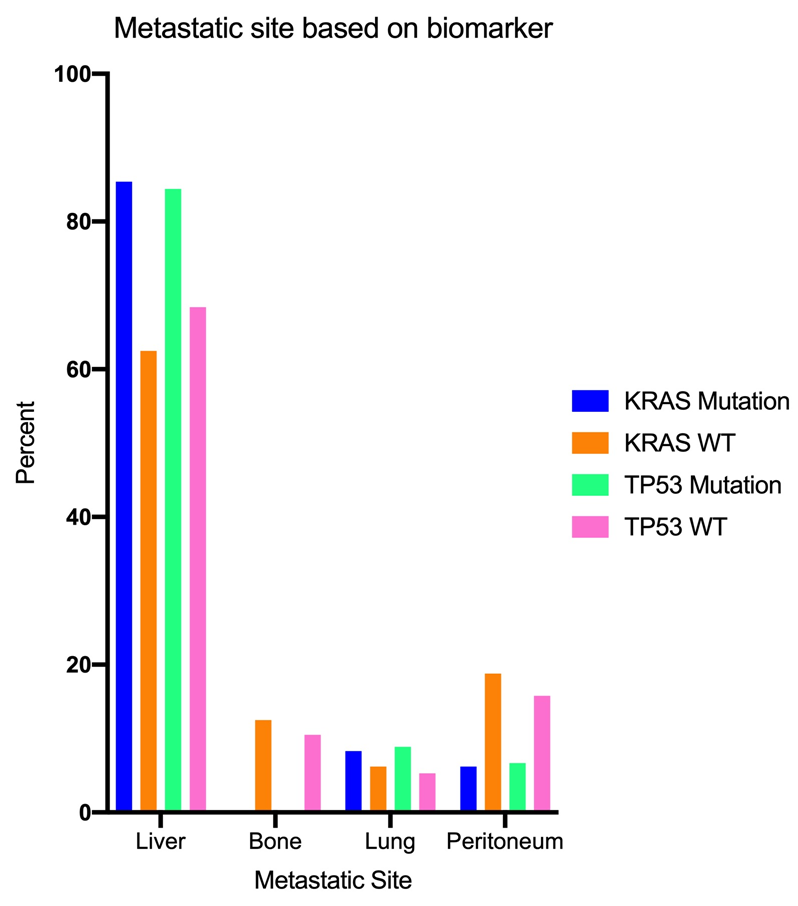


**Supplementary figure 3: Disease Stage Association With Somatic Alterations**

**
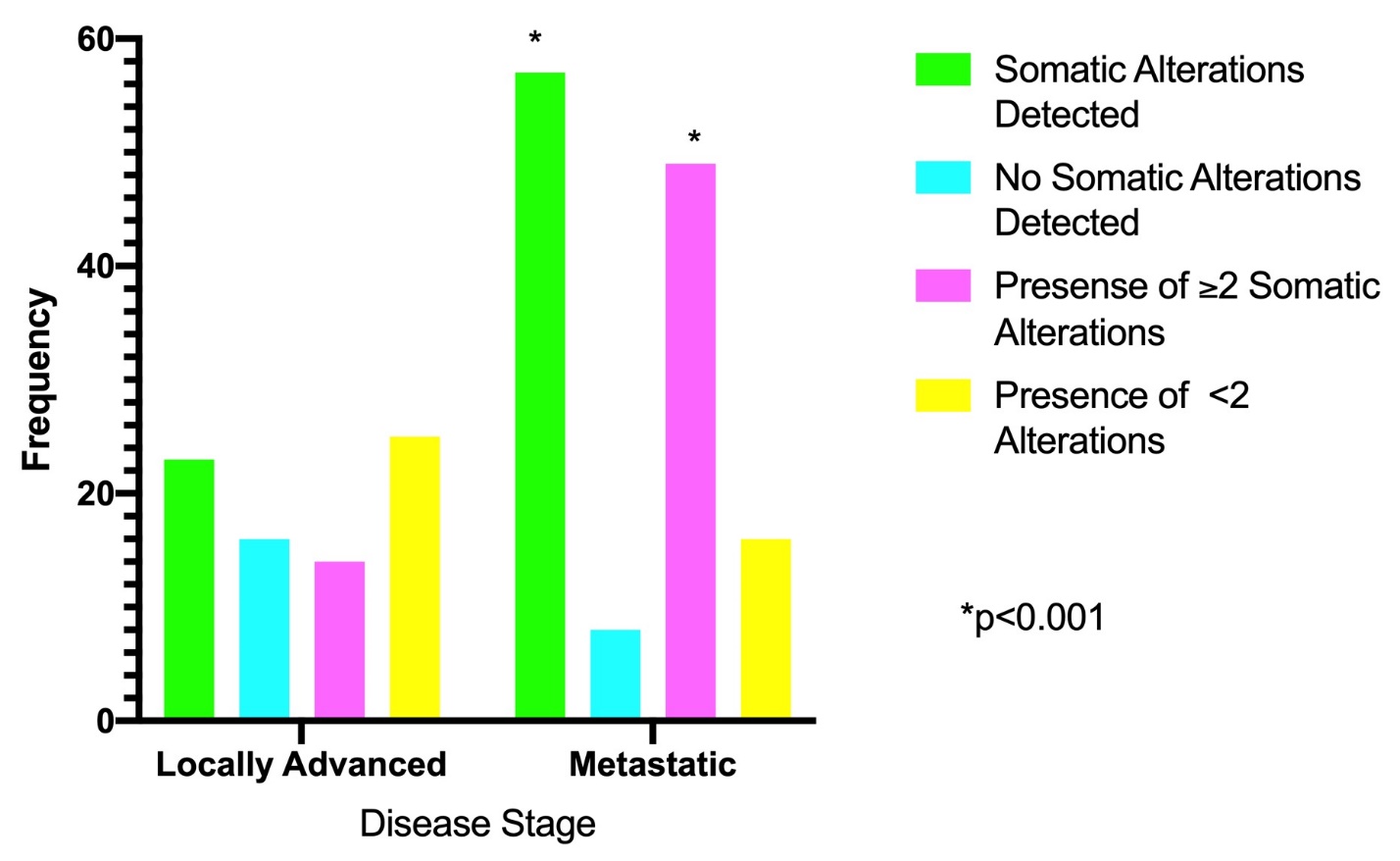
**

**Supplementary figure 4: ctDNA and response rate**

**
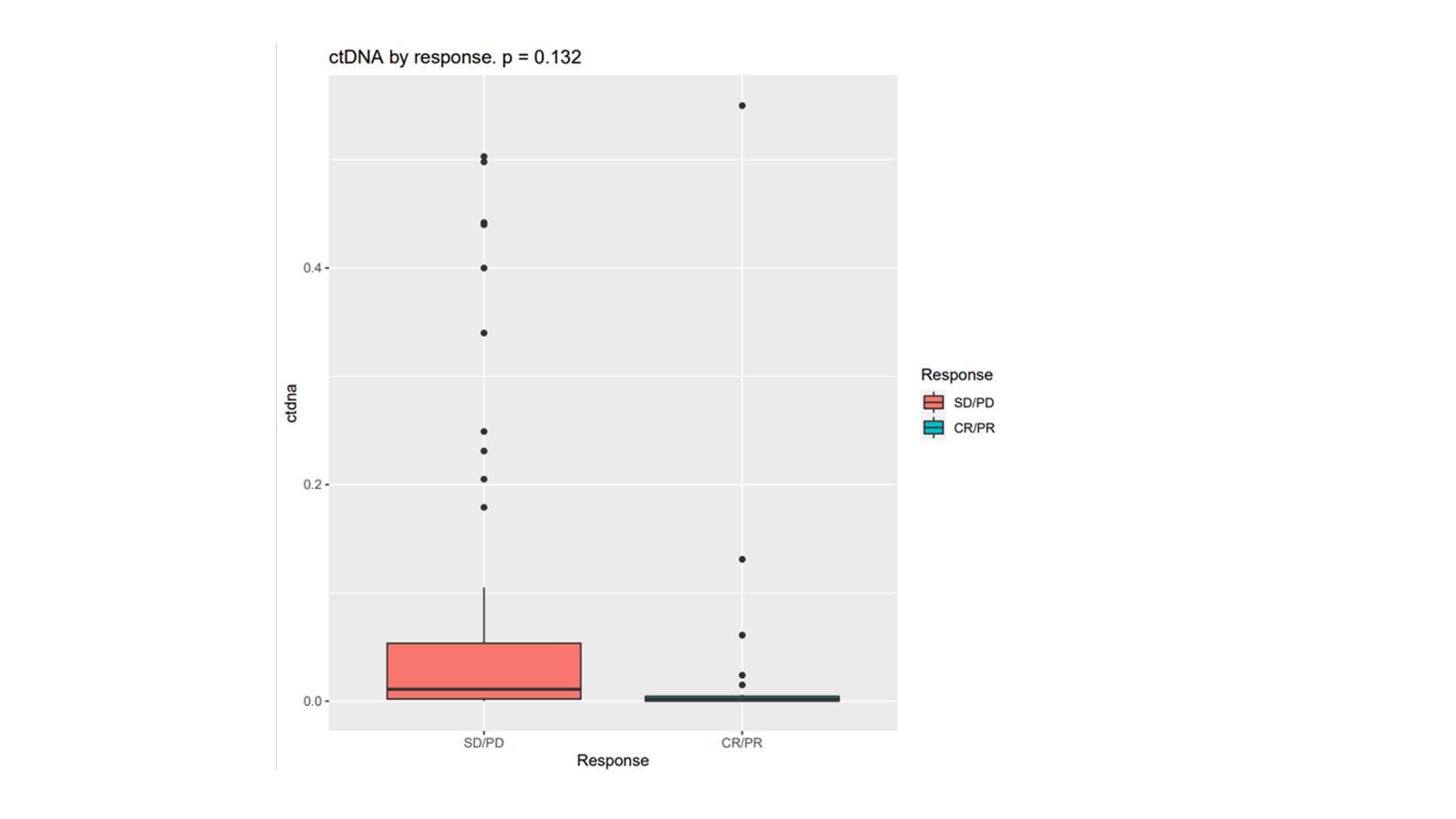
**

**Supplementary figure 5: ctDNA and disease control rate**

**
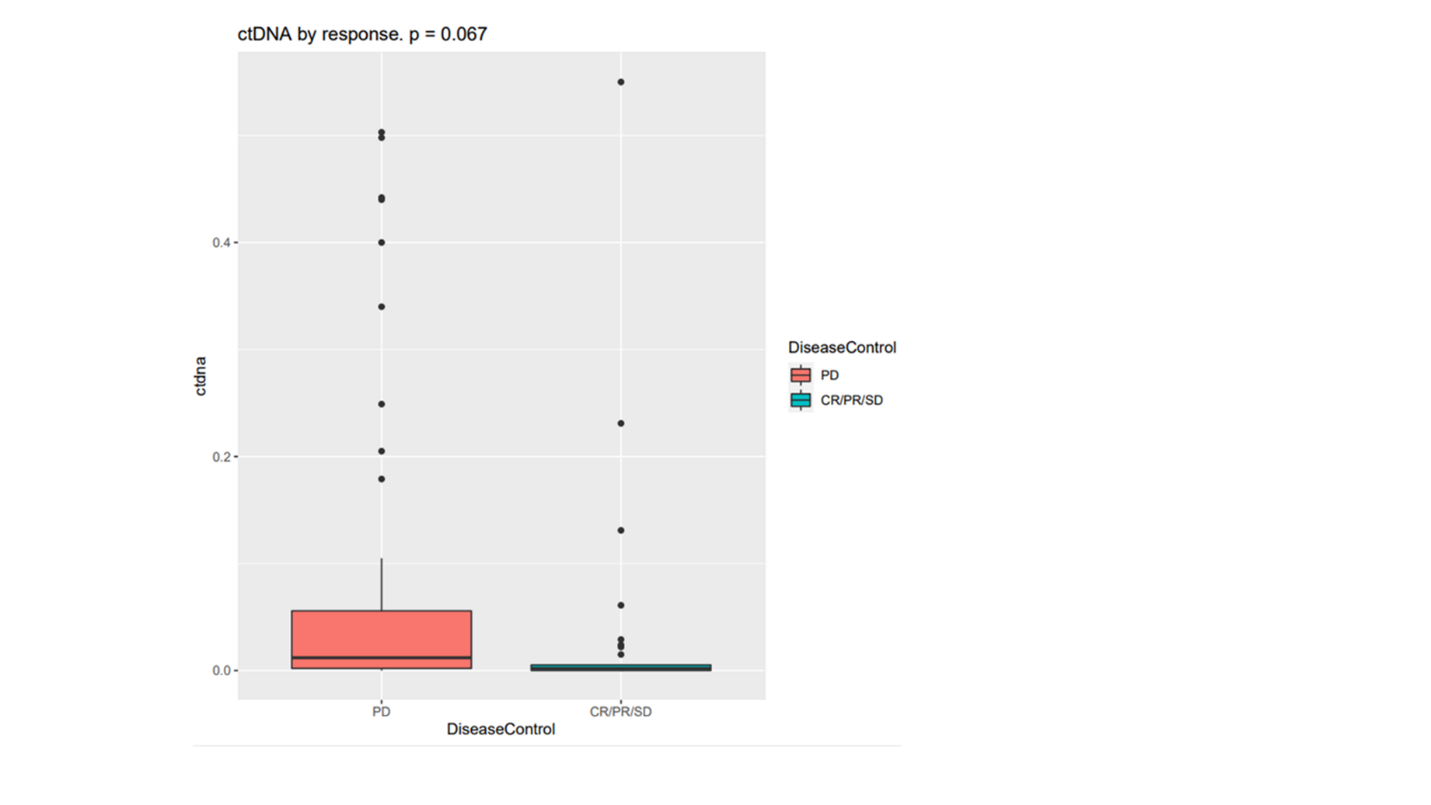
**
